# Longitudinal MR spectroscopy of neurodegeneration in multiple sclerosis with diffusion of the intra-axonal constituent *N*-acetylaspartate

**DOI:** 10.1016/j.nicl.2017.06.028

**Published:** 2017-06-22

**Authors:** Emily Turner Wood, Ece Ercan, Pascal Sati, Irene C.M. Cortese, Itamar Ronen, Daniel S. Reich

**Affiliations:** a Translational Neuroradiology Section, National Institute of Neurological Disorders and Stroke, National Institutes of Health, Bethesda, MD, USA; b Neuroimmunology Clinic, National Institute of Neurological Disorders and Stroke, National Institutes of Health, Bethesda, MD, USA; c C.J. Gorter Center for High Field MRI, Department of Radiology, Leiden University Medical Center, Leiden, Netherlands; d Radiology and Imaging Sciences, Clinical Center, National Institutes of Health, Bethesda, MD, USA

**Keywords:** MS, multiple sclerosis, DW-MRS, diffusion-weighted magnetic resonance spectroscopy, NAA, *N*-acetylaspartate, WM, white matter, HV, healthy volunteer, EDSS, Expanded Disability Scale Score, PASAT, Paced Auditory Symbol Addition Test, VOI, volume of interest, ICV, intracranial volume, T, Tesla, Multiple sclerosis, Diffusion-weighted magnetic resonance spectroscopy, Axonopathy, White matter

## Abstract

Multiple sclerosis (MS) is a pathologically complex CNS disease: inflammation, demyelination, and neuroaxonal degeneration occur concurrently and may depend on one another. Current therapies are aimed at the immune-mediated, inflammatory destruction of myelin, whereas axonal degeneration is ongoing and not specifically targeted. Diffusion-weighted magnetic resonance spectroscopy can measure the diffusivity of metabolites in vivo, such as the axonal/neuronal constituent *N*-acetylaspartate, allowing compartment-specific assessment of disease-related changes. Previously, we found significantly lower *N*-acetylaspartate diffusivity in people with MS compared to healthy controls (Wood et al., 2012) suggesting that this technique can measure axonal degeneration and could be useful in developing neuroprotective agents. In this longitudinal study, we found that *N*-acetylaspartate diffusivity decreased by 8.3% (p < 0.05) over 6 months in participants who were experiencing clinical or MRI evidence of inflammatory activity (n = 13), whereas there was no significant change in N-acetylaspartate diffusivity in the context of clinical and radiological stability (n = 6). As *N*-acetylaspartate diffusivity measurements are thought to more specifically reflect the intra-axonal space, these data suggest that *N*-acetylaspartate diffusivity can report on axonal health on the background of multiple pathological processes in MS, both cross-sectionally and longitudinally.

## Highlights

• Diffusion-weighted MR spectroscopy allows measurement of diffusion of the intraneuronal constituent, *N*-acetylaspartate (NAA).
• NAA diffusivity was measured in multiple sclerosis patients with and without inflammatory activity over 6 months.
• Multiple sclerosis patients with inflammatory activity had decreased diffusivity of NAA compared to stable MS patients.

## 1 Introduction

Axonal degeneration is an important direct cause of permanent disability in multiple sclerosis (MS) (Trapp et al., 1998), making its detection and measurement important for monitoring disease progression and therapeutic interventions. Indeed, whereas most people with MS initially experience a disease course characterized by episodic inflammatory relapses followed by extended periods of remission toward baseline function, many eventually suffer an underlying, gradual progression of neurological deficits in motor, sensory, and cognitive function. Even with the advent of disease-modifying therapies that decrease the incidence of inflammatory episodes, neurological function eventually declines in most cases (Kappos et al., 2010).

The pathophysiological basis of this decline in neurological function remains uncertain. However, examination of spinal cord tissue obtained at autopsy from MS patients has demonstrated progressive axonal neurofilament alterations, consistent with metabolic abnormalities, in chronic lesions with very low microglia/macrophage activity (Schirmer et al., 2011). Additionally, it has been found that neurodegeneration, assessed with magnetic resonance (MR) spectroscopy and brain volume measurements, may take place in the earliest stages of MS, in a “radiologically isolated” period before the onset of clinical signs and symptoms (Kappos et al., 2010, Stromillo et al., 2013), as well as in a “clinically isolated” period before people meet full diagnostic criteria for the disease (Bergsland et al., 2012, Schirmer et al., 2011, Wattjes et al., 2007).

In vivo MR techniques for measuring neurodegeneration in MS have mostly focused on the end result: atrophy, which can be measured in both brain and spinal cord on the time scale of years (Bermel and Bakshi, 2006, Fisher et al., 2008, Jones et al., 2013). These measurements demonstrate that the rate of atrophy is accelerated in MS (De Stefano et al., 2010) and that diminished brain volume measurements are correlated with clinical impairment (Gao et al., 2014, Rudick et al., 2009). Cross-sectional proton MR spectroscopy (^1^H MRS) studies in MS have demonstrated low concentrations of *N*-acetylaspartate (NAA), an in vivo neuroaxonal marker, within lesions, in extralesional “normal-appearing” white matter (WM) (Choi et al., 2007), and the whole brain, suggesting axonal loss (Ciccarelli et al., 2007, Davie et al., 1997, Filippi et al., 2003, Ge et al., 2004, Gonen et al., 2000, Inglese et al., 2003, Kirov et al., 2009, Leary et al., 1999, Narayana et al., 2004, Oh et al., 2004, Pelletier et al., 2003, Rovaris et al., 2005, Suhy et al., 2000, Tiberio et al., 2006). However, longitudinal studies in normal-appearing WM have not been able to detect declining NAA concentration in patients with MS over a 2–3 year span (Kirov et al., 2012, Sajja et al., 2008, Tiberio et al., 2006).

Diffusion tensor imaging (DTI) has been heavily applied in MS and animal models of MS, with conflicting results. Most DTI studies in humans in vivo have demonstrated increased water diffusivity and decreased water fractional anisotropy (FA) in both lesions and normal-appearing WM (Bammer et al., 2000, Evangelou et al., 2000, Reich et al., 2010, Roosendaal et al., 2009, Werring et al., 1999). Fink et al. compared DTI measures of regions derived from tractography and brain volume analysis to distinguish the types of information contributed by these techniques (Fink et al., 2010). Their analysis showed that whereas both volumes and DTI values, such as FA, are loosely associated with composite measures of disease progression, such as lesion load and the Expanded Disability Status Scale (EDSS), they are not specific for the underlying biological process, e.g. inflammation or neurodegeneration (Samann et al., 2012).

In a previous cross-sectional study, we measured diffusion of NAA in the human normal-appearing corpus callosum on a 7-Tesla (T) MRI scanner, comparing 15 people with MS and 14 healthy controls (Wood et al., 2012). The corpus callosum is a good location for studying axonal degeneration from anatomical, functional, and technical standpoints: it is the largest WM structure; it is involved in multiple sensory, attentional, and cognitive processes; and the fibers are quasi-coherent in orientation. We found that NAA *parallel diffusivity* (λ), i.e. diffusivity along the axonal propagation direction, was on average significantly diminished in MS and, importantly, inversely correlated with both DTI measures, i.e. mean diffusivity (MD) and fractional anisotropy (FA), and with clinical severity (EDSS). Our findings provided preliminary evidence that diffusion-weighted MR spectroscopy (DW-MRS) of NAA can distinguish axonopathy from other processes such as inflammation, vasogenic edema, demyelination, and gliosis. Through subsequent studies with healthy volunteers, we demonstrated that axonal modeling can be used to yield NAA diffusivity values that more accurately reflect the cytosolic diffusion coefficient of NAA while minimizing the confounding effects of inter-subject differences and large spectroscopy voxels (Ronen et al., 2014).

In MS, axonal degeneration plays out over time. In this study, therefore, we asked whether the DW-MRS technique is capable of detecting axonopathy in WM tissue over a period of six months. For this, we evaluated a new cohort of MS patients that was divided into groups with stable and active disease courses, and a third group of healthy controls allowed us to add cross-sectional data at the two time points. In the process, we also established that DW-MRS is feasible in a clinical setting with a 3 T scanner.

## 2 Materials and methods

### 2.1 Participants

The National Institutes of Health Institutional Review Board approved this study. All participants gave informed consent. Participants were neurologically evaluated in the Neuroimmunology Clinic, National Institute of Neurological Disorders and Stroke, NIH, Bethesda, Maryland, USA.

The MS cohort was recruited from the Neuroimmunology Clinic and consisted of a wide range of cases – from stable cases with no new lesions for 1–10 years (based on existing prior MRI scans) to cases with high disease activity where new T_2_ lesions had formed in the 6 months before or during the study. For this study, 19 participants who met McDonald criteria for MS (Polman et al., 2011) were scanned. At recruitment, 9 were stable – they had not had a relapse or new T_2_ lesion for at least 1 year. Three of these had a new T_2_ lesion during the study so were reclassified as active. Therefore, at study conclusion, there were 6 stable cases and 13 active cases. Three stable and 10 active cases were on disease-modifying therapies.

Healthy volunteers (HVs) were recruited from the NIH Clinical Research Volunteer Program. The HVs had no history of neurological conditions. Each HV was examined by a neurologist and underwent clinical MRI scans that were within normal limits. HVs were compensated for taking part in the study.

All MS cases were scanned at baseline (“month 0”) and month 6. Most active MS cases were also scanned at month 3 in order to more closely follow disease activity. Five of the 6 HVs were scanned twice, with scans separated by 1–14 days. For all repeat scans, volumes of interest were positioned to match, as much as possible, the original placement.

MS cases were assessed at month 0 and month 6 with the EDSS, Paced Auditory Symbol Addition Test (PASAT, 3-second version), 9-Hole Peg Test, and 25-Foot Timed Walk. By design, clinical data were obtained within 30 days of MRI acquisition. The average time lag between MRI and clinical exam was 2 days for both stable and active patients, with 32 of 38 scans obtained within 1 day of clinical exam and the remaining occurring within 6, 14, 27, 28, 30 and 30 days of the clinical exam.

During each scan session, structural and DW-MRS scans were acquired for all participants. All scans were acquired on 3 T Philips Achieva scanners (Philips Medical Systems, Best, The Netherlands) in the NIH Clinical Center Radiology and Imaging Sciences Department. These scanners have gradients with a maximum amplitude of 80 mT/m and a slew rate of 100 T/m/s, quadrature volume transmit coils, and 8-channel receive head coils.

### 2.2 Structural image acquisition and processing

All subjects were scanned with the following order of sequences: 3D T_1_-weighted gradient echo, DTI, two DW-MRS volumes of interest (VOIs), and a T_2_-weighted FLAIR. Lastly, gadolinium-enhanced T_1_-weighted scans (see below) were acquired for all active MS cases and for stable cases at the discretion of the clinician.

3D T_1_-weighted gradient echo images were acquired with an inversion-prepared turbo field echo (TFE) sequence and were used for positioning of the VOI in the DW-MRS experiments and for tissue segmentation in the post-processing stage. Imaging parameters were: field of view (anterior-posterior × foot-head × right-left) = 240 × 240 × 180 mm^3^, 1 mm isotropic resolution, TR/TE = 7.00 ms/3.15 ms, TI = 874.2 ms, SENSE = 2(AP) × 3(RL), and total scan time = 5.30 min + delay for scanner preparation. Whole brain DTI images were acquired using single-shot 2D spin-echo echo-planar imaging. DTI parameters were: field of view = 224 × 224 × 120 mm^3^, 2 × 2 × 2 mm^3^ isotropic resolution, TR/TE = 7487 ms/85 ms, 32 diffusion weighting directions with b = 800 s/mm^2^, SENSE = 3(AP), and total scan time = 5.50 min.

T_2_-weighted Fluid Attenuated Inversion Recovery (FLAIR) images were acquired for all patient scan sessions using a 3D–FLAIR-VISTA (volume isotropic turbo spin-echo acquisition) sequence with parameters: field of view = 240 × 240 × 180 mm^3^, 1 mm isotropic resolution, TR/TE = 4800 ms/365 ms, TI = 1600 ms, SENSE = 2.6(AP) × 2(RL), and total scan time = 6.00 min. FLAIR images were acquired just prior to acquisition of the final contrast-enhanced T_1_-weighted volume. For this reason, gadolinium contrast was administered during the FLAIR scan: a single dose (0.1 mmol/kg) of gadobutrol (Gadavist; Bayer Healthcare, Leverkusen, Germany) was injected by power injector (Medrad, Warrendale, PA) over a period of 60 s.

T_1_-weighted, FLAIR and DTI volumes were processed in MIPAV (McAuliffe et al., 2001) (Medical Image Processing, Analysis and Visualization) and JIST (Lucas et al., 2010) (Java Image Science Toolbox). The T_1_-weighted and FLAIR images within an acquisition were rigidly registered to each other and subsequently to the Montreal Neurological Institute (MNI) atlas with the Optimized Automatic Registration (OAR) algorithm (Jenkinson and Smith, 2001), inhomogeneity-corrected using N3,(Sled et al., 1998) and skull-stripped with the Simple Paradigm for Extra-Cerebral Tissue Removal (Carass et al., 2011) (SPECTRE). Tissue segmentation into WM, gray matter (GM), deep gray structures, cerebrospinal fluid (CSF), and lesions was performed with Lesion TOpology-preserving Anatomy-Driven Segmentation (Bazin and Pham, 2008, Shiee et al., 2010) (Lesion-TOADS) (IACL, Johns Hopkins University). Lesion-TOADS is specifically adapted for segmenting the MS brain by using a probability-based algorithm to account for the likelihood of lesions in WM. It utilizes both the T_1_-weighted and FLAIR image intensities, in conjunction with empirically derived statistical and topological brain atlases, to assign tissue type. We have reported that Lesion-TOADS-derived brain structure volumes are more strongly associated with physical impairment in MS than those derived from SIENAX (Shiee et al., 2010, Shiee et al., 2012, Smith et al., 2002) (FMRIB, Oxford University). For HVs, segmentation was performed with TOADS without a lesion category. Two HVs did not have FLAIR images. Brain volume values are reported as a percentage of intracranial volume (ICV), and lesion load is calculated as a percentage of WM volume.

For the whole brain DTI images, the individual diffusion-weighted volumes were eddy-current and motion corrected. The b = 0 s/mm^2^ image was registered using affine transformation to the T_1_-weighted volume in MNI space with appropriate gradient-vector rotation. From this, the diffusion tensor was estimated and diagonalized for each voxel to yield maps of the primary eigenvector, fractional anisotropy (FA), mean diffusivity (MD), parallel diffusivity (λ _||_ = λ_1_), and perpendicular diffusivity (λ _⊥_ = (λ_2_ + λ_3_)/2). The WM VOI mask attained from segmentation of the T_1_-weighted volume was then applied to these images to obtain eigenvectors and diffusion values corresponding to the acquired DW-MRS VOI.

### 2.3 DW-MRS volume of interest & acquisition

In each session, two DW-MRS VOIs were acquired from corpus callosum WM. The corpus callosum is a good location for studying axonal degeneration from anatomical, functional, and technical standpoints. The corpus callosum is centrally located, easily distinguished on conventional MR images, and contains axons that connect corresponding cortices of the right and left hemispheres. These fibers are important for midline fusion of sensory information, coordinating interhemispheric processing, and attentional control (Aboitiz and Montiel, 2003, Banich, 1998). At the midline, the corpus callosum has enough quasi-coherently oriented fibers to fill the relatively large voxel that is required to generate adequate signal for diffusion weighted spectroscopy experiments to be performed. The corpus callosum is frequently damaged in MS, and this damage is linked to disability (Hasan et al., 2012b, Lenzi et al., 2007, Ozturk et al., 2010, Yaldizli et al., 2011). Alterations in quantitative MRI measures, such as DTI and relaxometry in the corpus callosum, are associated with whole brain measures, such as lesion load, and functional readouts, such as the EDSS score (Hasan et al., 2012a, Reich et al., 2010).

**Fig. 1:**
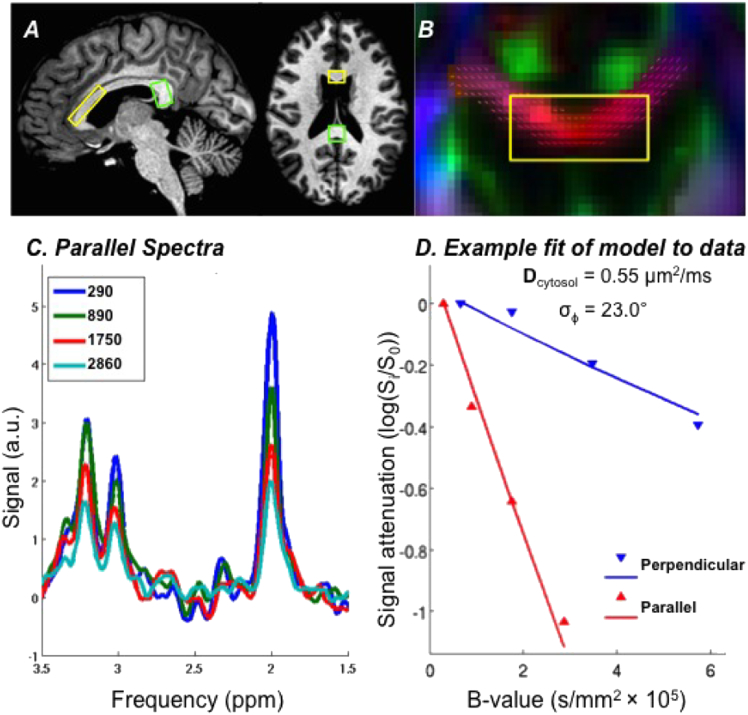
Voxel-of-interest localization, spectra and model fit. A. DW-MRS voxel-of-interest (VOI) placement on anterior corpus callosum (yellow) and splenium (green). B. Coronal view of anterior corpus callosum on DTI color map overlaid with the primary eigenvector (VOI in yellow, color denotes direction of primary eigenvector: red = right-left, green = anterior-posterior, blue = foot-head). C. Diffusion weighted spectra acquired parallel [1,0,0] to the corpus callosum fibers at 4 b-values (s/mm^2^). D. Triangles: Logarithm of the diffusion-weighted signal measured from one active MS case with diffusion weighting applied along the parallel ([1,0,0], red) and perpendicular ([0,-1,1], blue) directions as a function of b-value (s/mm^2^). Lines: Model fit, which yields cytosolic diffusivity **D**_cytosol_ (σ_φ_ = the standard deviation of the axonal angular dispersion).

For all participants, one VOI measured (anterior-posterior × foot-head × right-left) 30 × 15 × 8 mm^3^ (3600 mm^3^) and was placed on the genu and anterior body of the corpus callosum (yellow box in Fig. 1A). The second VOI was placed on either the posterior body (16 MS, 5 HV; same dimensions as anterior corpus callosum volume) or splenium (2 MS active, 1 MS stable, 1 HV; 12 × 15 × 18 mm^3^ (3240 mm^3^)) of the corpus callosum (green box in Fig. 1A demonstrates splenium placement). The splenium location was chosen when the posterior body was very thin or curved such that the VOI could not adequately contain it, and our processing scheme specifically accounts for varying locations across the corpus callosum.

The PRESS (Point Resolved Spectroscopy) sequence was chosen as the base spectroscopic sequence for these single volume DW-MRS experiments and was supplemented with a bipolar diffusion-weighting scheme for minimization of eddy currents and of cross-terms with background and imaging gradients. See Kan et al. (2012) for details and schematic. For the diffusion weighting, two directions were chosen for all scans: (1) a pure right-left direction in the VOI frame, which is mostly parallel to the direction of the callosal fibers; and (2) a direction perpendicular to the callosal fibers, forming a 45° angle between the anterior-posterior axis and the inferior-superior axis of the VOI.

These gradient directions can be denoted in the VOI coordinates as [1,0,0] and [0,− 1,1]. The position of the gradient directions with respect to the VOI is shown in Fig. 1A. In all experiments, the center frequency was set to that of the NAA singlet peak at 2.0 ppm. Water suppression was achieved using two frequency-selective excitation pulses centered at the water resonance frequency, followed by dephasing gradients. The water suppression was “de-optimized” for the diffusion-weighting conditions to allow sufficient residual water signal for later use in the post-processing stage for zero-order phase correction and frequency-drift correction of individual spectra prior to spectral averaging. A wired peripheral pulse unit was used for cardiac synchronization of the DW-MRS acquisition to minimize signal fluctuations due to cardiac pulsation (Upadhyay et al., 2007). Pencil-beam shimming was applied up to second order, resulting in a typical NAA singlet linewidth of 7 Hz. Following each scan, a shorter scan with identical VOI position and diffusion conditions was performed with the center frequency set at the water resonance frequency and without water suppression. This scan was subsequently used for eddy-current correction.

The DW-MRS parameters were: TE = 110 ms, TR = 2 cardiac cycles (about 2000 ms), trigger delay = 200 ms, number of time-domain points = 1024, spectral width = 1500 Hz, gradient duration (δ) = 22 ms, bipolar gap = 20 ms, diffusion time (Δ) = 55 ms with 4 different gradient amplitudes resulting in b-values of 290, 890, 1750, and 2860 s/mm^2^ in the [1,0,0] direction and 650, 1760, 3460, and 5730 s/mm^2^ in the [0,-1,1] direction. 48 spectra were collected for each diffusion condition (2 directions, 4 b-values), plus an additional 48 for the highest gradient in each direction. Total DW-MRS scan time ranged from 16 to 24 min, depending upon heart rate.

### 2.4 DW-MRS data processing

Post-processing of the DW-MRS data was performed using custom Matlab routines (Mathworks, Natick MA). Following DW-MRS data acquisition, the individual spectra were first corrected for zero-order phase fluctuations and frequency drift according to the remaining water peak, and then eddy-current corrected using the unsuppressed water acquisition (Upadhyay et al., 2007). Spectra were averaged within condition, and then the remaining water peak was removed using a Hankel Singular Value Decomposition (HSVD) method. These spectra were quantified using LCModel using a simulated appropriate basis set (Provencher, 1993).

The LCModel output was used as an input to a modeling routine that calculates the *intra-axonal*, or *cytosolic*, diffusivity of NAA. This procedure is designed to minimize the variability in the DW-MRS measurements introduced by macroscopic factors, such as the position of the VOI within the WM tract, the main direction of the tract with respect to the DW gradients, and the macroscopic curvature of the tract within the VOI. The model, described in Ronen et al. (2013), assumes that the diffusion-weighted NAA signal can be decomposed into the contributions from the diffusion of NAA parallel and perpendicular to the axons. It also uses the angles between the main eigenvectors of the DTI data within the DW-MRS VOI and the diffusion-weighting gradient directions to account for the macroscopic curvature of the corpus callosum within the VOI. The diffusion-weighted data in both directions are fitted simultaneously to the model using two variables: **D**_cytosol_ the cytosolic diffusion coefficient of NAA, and σ_φ_, the standard deviation of the axonal angular dispersion (Fig. 1D). Importantly, **D**_cytosol_ is independent of the tract geometry within the VOI and thus mostly reflects the effects of the cytosolic medium, e.g. viscosity, molecular crowding and the integrity of intra-axonal structures, on the diffusion of NAA inside the axons.

### 2.5 Statistical analysis

Between-groups analyses – stable versus active or MS versus HV – of demographic, cross-sectional, and longitudinal difference measures were tested with mixed-effects models that account for the random effects of each participant contributing 2 VOIs to the data set. In this way, cross-sectional analyses were performed for each time point separately. Additionally, the data for all time points together were analyzed with a mixed-effects model accounting for both the effect of multiple time points from the same VOI and multiple VOIs from the same participant. Data were assessed for normality based on skewness and kurtosis. NAA **D**_cytosol_ and all DTI measures were mesokurtic, whereas ICV was platykurtic. NAA **D**_cytosol_ and FA were approximately symmetric. MD, λ _||_, λ _⊥_, and ICV were negatively skewed. As the MS and HV groups were not balanced for sex, sex was a covariate for all MS versus HV analyses.

Longitudinal analyses consisted of calculating the difference between measures at month 6 and baseline (Δ = *t*_mo__6_ − *t*_mo 0_; a positive difference value represents an increase at month 6). For comparison, HV differences are shown between the first and second scans. A one-sample Student's *t*-test was used to test the null hypothesis Δ = 0. Otherwise, mixed-effects models were used to account for the random effect of multiple VOIs. Linear regression was used to characterize associations between DW-MRS measures, DTI measures, brain volumes, age, disease duration (measured as time since onset of symptoms attributable to MS), and EDSS. The regression coefficient (*RC*), coefficient of determination (*R*^2^), and *p* value are reported. Changes in EDSS and 25-Foot Timed Walk were evaluated with the Mann-Whitney *U* test.

Power and sample size calculations for DW-MRS with a similar set of b-values at 3 T were reported previously (Wood et al., 2015). In the present study, the goal was to establish whether DW-MRS may be worth pursuing further as a marker of neurodegeneration. Therefore, more type 1 errors were considered tolerable, and there was no planned correction for multiple comparisons. Statistical analyses were performed with GraphPad Prism version 6.0b for Mac OS X, GraphPad Software, San Diego, California, USA and STATA release 11, StataCorp, College Station, Texas, USA.

## 3 Results

### 3.1 Patient scans and demographics

Baseline demographics for all participants are shown in Table 1. There was no significant difference between the stable and active MS groups for age, sex, disease duration, EDSS, PASAT score, 25-Foot Timed Walk time and 9-Hole Peg Test.

**Table. 1:** Participant demographics at baseline.

		Stable MS (n = 6)	Active MS (n = 13)	Stable vs. Active p-value	Healthy volunteers (n = 6)
Age, years	Mean (SD) range	48 (13) 30–69	41 (12) 20–62	0.26	48 (9) 40–62
Sex	Number of women	4	6	0.63	1
Clinical phenotype	RR, PP	6 RR	12 RR, 1 PP		
Disease duration, years	Mean (SD)	9 (5) 3–14	6 (4) 1–12	0.14	
EDSS	Median range	1.5 0–2	1.5 0–6.5	0.41	
PASAT score	Mean (SD) range	51 (8) 41–60	51 (10) 25–60	0.92	
25-foot walk time, seconds	Mean (SD) range	5.0 (1.1) 4.0–7.1	4.6 (1.5) 3.2–8.3^a^	0.58	
9-hole peg test, seconds	Mean (SD) range	20 (2.0) 17–23	20 (2.7) 15–24^b^	0.81	

a n = 11, as 2 MS cases were excluded: 1 uses an assistive device for walking (25WT = 117.8 s), and 1 had an injury requiring a temporary assistive device.

b n = 8, as 5 participants declined to perform.

Spectra were of good quality (LCModel NAA estimates with Cramer-Rao Lower Bounds < 15%) for 93 of 96 DW-MRS volume acquisitions (97%). One stable-MS and 1 active-MS patient could only tolerate acquisition of one CC VOI resulting in 11 stable-MS and 25 active-MS collected at baseline. There was month-6 follow-up for 10 of the stable-MS and 22 of the active-MS DW-MRS VOIs (89%). As there was no statistical difference found in DW-MRS measurements between anterior and posterior corpus callosum VOIs, these groups were combined. Therefore, in total there were 10 stable-MS and 22 active-MS DW-MRS volumes with both baseline and month-6 data for longitudinal analysis. While 6 HVs were scanned twice at baseline, the second acquisition for one HV was unusable due to motion artifacts. Therefore, there were 10 HV DW-MRS volumes acquired at two time points. As planned for patients with known active disease at baseline, there were 11 MS cases with month-3 scans. For the cross-sectional analysis, all available time points (baseline, month 3, and month 6) were averaged for each DW-MRS volume, resulting in 10 HV, 10 stable-MS and 26 active-MS DW-MRS measurements. All DTI-acquired water diffusivity measures are from the same corpus callosum volumes of interest as the corresponding DW-MRS measurements. (See Table 2.)

**Table 2:** Data for all time points.

		Stable MS (n = 6)	Active MS (n = 13)	Stable vs. Active p-value	All MS (n = 19)	HVs (n = 6)	MS vs. HV p-value
NAA **D**_cytosol_ (μm^2^/ms)	*All t*	0.46 (0.07)	0.49 (0.07)	ns	0.48 (0.07)	0.52 (0.05)	0.015
	*t*_mo 0_	0.45 (0.07)	0.50 (0.07)	0.035*	0.49 (0.07)		
	*t*_mo 3_		0.50 (0.08)				
	*t*_mo 6_	0.48 (0.08)	0.47 (0.05)	ns	0.47 (0.06)		
DTI MD (μm^2^/ms)	*All t*	1.24 (0.15)	1.23 (0.15)	ns	1.24 (0.15)	1.10 (0.08)	0.004
	*t*_mo 0_	1.21 (0.12)	1.24 (0.16)	ns	1.23 (0.15)		
	*t*_mo 3_		1.24 (0.16)				
	*t*_mo 6_	1.27 (0.18)	1.22 (0.12)	ns	1.24 (0.14)		
DTI FA	*All t*	0.55 (0.06)	0.53 (0.06)	ns	0.53 (0.06)	0.57 (0.04)	ns
	*t*_mo 0_	0.55 (0.05)	0.53 (0.06)	ns	0.54 (0.06)		
	*t*_mo 3_		0.53 (0.06)				
	*t*_mo 6_	0.54 (0.07)	0.53 (0.05)	ns	0.53 (0.06)		
DTI λ _||_ (μm^2^/ms)	*All t*	2.01 (0.14)	1.97 (0.15)	ns	1.98 (0.14)	1.85 (0.08)	< 0.0001
	*t*_mo 0_	1.98 (0.11)	1.99 (0.15)	ns	1.98 (0.14)		
	*t*_mo 3_		1.97 (0.17)				
	*t*_mo 6_	2.04 (0.16)	1.96 (0.12)	ns	1.98 (0.14)		
DTI λ _⊥_ (μm^2^/ms)	*All t*	0. 85 (0.17)	0.86 (0.16)	ns	0.86 (0.16)	0.73 (0.10)	0.0003
	*t*_mo 0_	0.81 (0.14)	0.87 (0.17)	ns	0.85 (0.16)		
	*t*_mo 3_		0.87 (0.17)				
	*t*_mo 6_	0.89 (0.19)	0.85 (0.13)	ns	0.86 (0.15)		
%ICV (%)	*All t*	79.3 (3.3)	79.9 (1.9)	ns	79.8 (2.3)	82.0 (1.4)	0.035
	*t*_mo 0_	79.2 (3.3)	79.9 (1.9)	ns	79.7 (2.4)		
	*t*_mo 3_		80.1 (2.2)				
	*t*_mo 6_	79.3 (3.5)	79.7 (1.8)	ns	80.0 (2.4)		
Lesion load (ml |%WM)^a^	*All t*	6.5 | 1.3	8.5 | 1.7	ns	8.1 | 1.6	0	NA
	*t*_mo 0_	6.6 | 1.4	8.9 | 1.8	ns	8.2 | 1.6		
	*t*_mo 3_		8.7 | 1.8				
	*t*_mo 6_	6.3 | 1.3	8.0 | 1.6	ns	7.5 | 1.5		
VOI lesion load (mm^3^)	*All t*	30 (59)	45 (62)	ns	42 (62)	0	NA
	*t*_mo 0_	37 (65)	50 (69)	ns	46 (67)		
	*t*_mo 3_		51 (66)				
	*t*_mo 6_	22 (53)	35 (53)	ns	31 (53)		

Means with standard deviations in parentheses.

a For lesion load, values reported are mean absolute volume in milliliters and percent of white matter volume (without SD).

### 3.2 Cross-sectional analysis

**Fig. 2:**
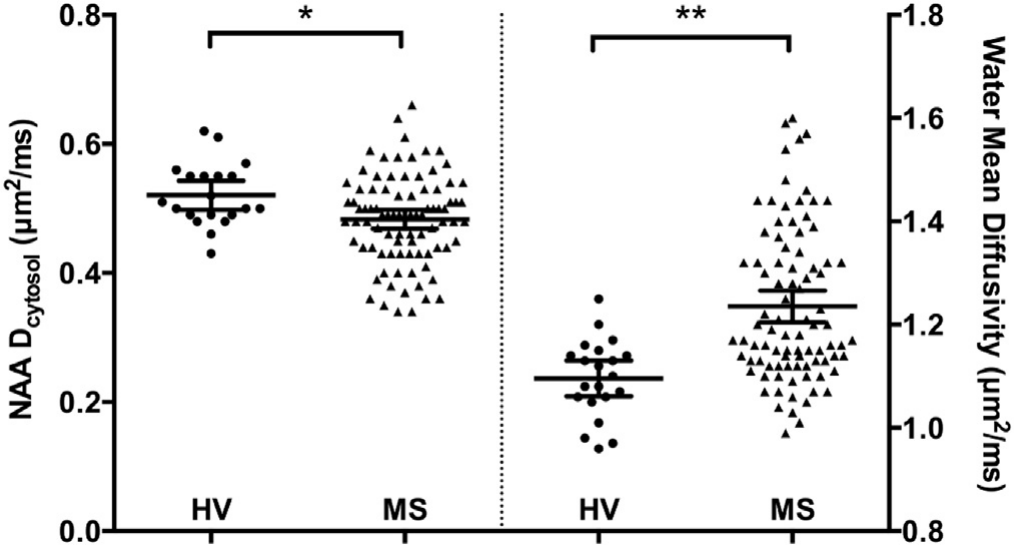
Cytosolic NAA diffusivity and water mean diffusivity for healthy volunteers and MS cases. *p < 0.05. Bars show group mean and 95% confidence interval.

In line with our previous findings of water and NAA diffusivity measures in the corpus callosum in a cross-sectional study, MS patients had lower average NAA **D**_cytosol_ (MS mean **D**_cytosol_ = 0.48 μm^2^/ms, HV mean **D**_cytosol_ = 0.52 μm^2^/ms, p = 0.015) and higher average water MD (MS mean MD = 1.24 μm^2^/ms, HV mean MD = 1.10 μm^2^/ms, p = 0.004) compared to healthy volunteers (Fig. 2) (Wood et al., 2012). In this cohort, NAA diffusivity measures were not significantly correlated with any water diffusivity measures.

Brain volume – estimated as a percentage of total intracranial volume – was decreased in patients with MS compared to HVs (MS %ICV = 79.8, HV %ICV = 82.0, *p* = 0.035) and negatively correlated with age for both MS cases (*RC* = − 0.11%ICV/year, *R*^2^ = 0.36, p < 0.001) and HVs (*RC* = − 0.12%ICV/year, *R*^2^ = 0.46, p = 0.01). Water MD correlated negatively with %ICV for MS cases (*RC* = − 0.03 MD/%ICV, *R*^2^ = 0.36, p < 0.001) and HVs (*RC* = − 0.02 MD/%ICV, *R*^2^ = 0.36, p < 0.05).

The segmentation method used here, Lesion-TOADS, was designed for use in MS and is configured such that only lesions within WM structures are segmented. Measurements of lesion load, both as percentage of total WM (%WM) and as a volume (μL) within the DW-MRS volumes of interest, demonstrated no differences between active and stable MS groups. In the MS group, lesion load (%WM) was negatively correlated with NAA **D**_cytosol_ and water FA and positively correlated with water MD, λ _⊥_, and λ _||_ (Supplementary Fig. 1). Disease duration was not statistically different between stable and active MS groups, although there was a trend toward those with the shortest disease duration being in the active group (*ns*, Supplementary Fig. 2A).

### 3.3 Longitudinal analysis

**Fig. 3:**
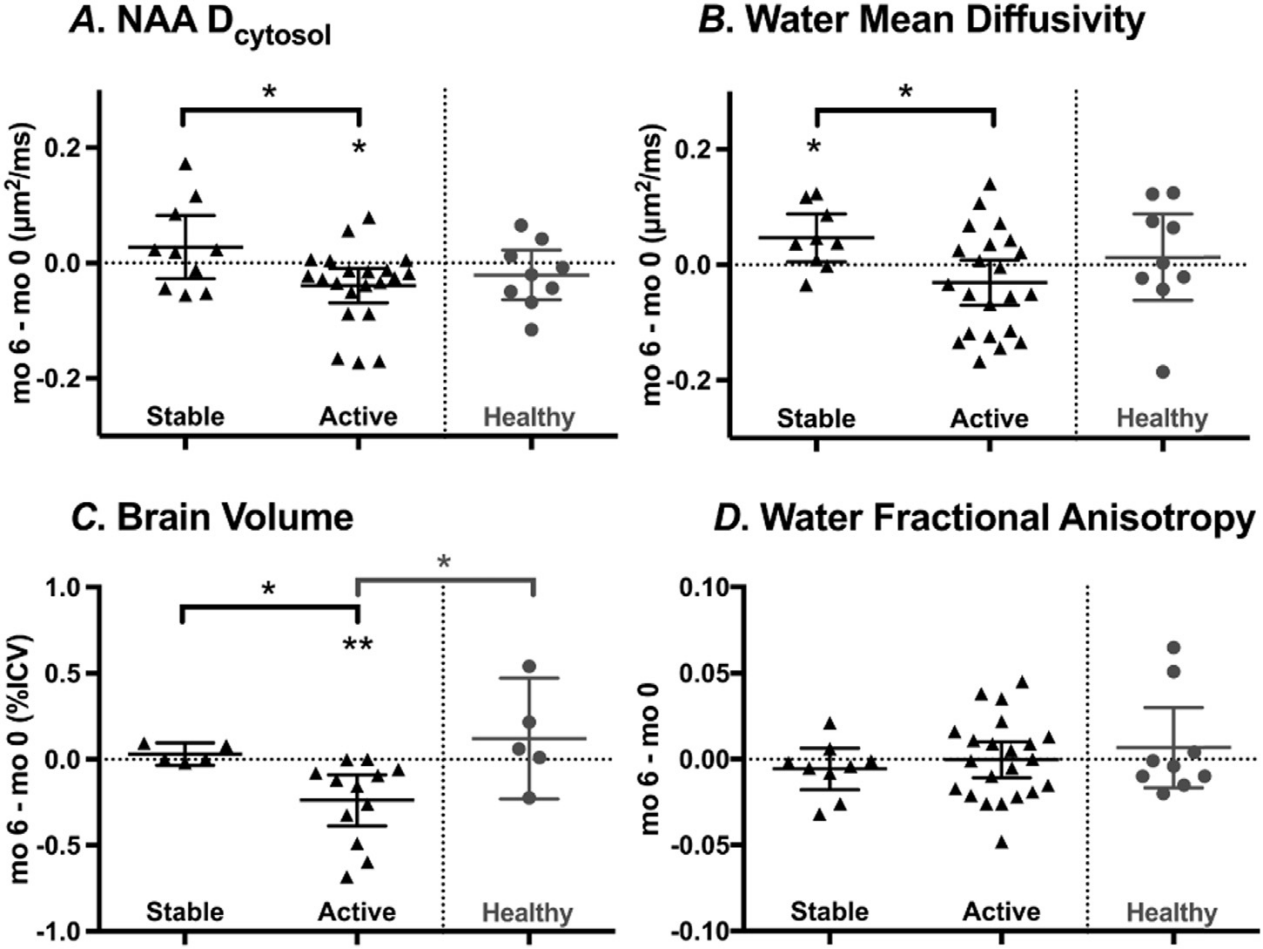
Difference values (Δ) between baseline and month 6 for stable and active MS cases. A. Cytosolic NAA diffusivity, *p < 0.05 for stable ≠ active, *p < 0.05 for active ≠ 0. B. Water mean diffusivity (MD), *p < 0.05 for stable ≠ active, *p < 0.05 for stable ≠ 0. C. Brain volume (% = total brain/total intracranial volume), *p < 0.05 for stable ≠ active, **p < 0.005 for active ≠ 0, *p < 0.05 for active ≠ healthy. D. Water fractional anisotropy, ns. For MS patient, Δ = t_mo__6_ − t_mo 0_ such that a positive difference value represents an increase at month 6. For healthy controls, scans were < 39 days apart, and the group mean with 95% confidence interval is shown for reference.

To assess changes in diffusivity values between baseline and month 6, difference values were calculated (Δ = *t*_mo__6_ − *t*_mo 0_) for each DW-MRS VOI; a positive value expresses an increase in a measurement over 6 months. These difference values were then compared between the stable and active MS groups. HVs were scanned twice within 2 weeks, not at baseline and 6 months; therefore, HV difference values are not directly comparable to MS values and are shown only for reference (Fig. 3). For HVs, the average difference between scans for NAA diffusivity, water diffusivity, and brain volume measures were not statistically different from zero nor were they different from stable or active MS groups except for brain volume with active MS (p = 0.02).

**D**_cytosol_ difference values distinguished the stable and active MS groups – stable mean Δ**D**_cytosol_ = 0.03 μm^2^/ms, active mean Δ**D**_cytosol_ = − 0.04 μm^2^/ms (p = 0.03) – primarily based on lower MS **D**_cytosol_ values in active cases at month 6 (one-sample *t*-test of active mean Δ**D**_cytosol_ ≠ 0, p = 0.01) (Fig. 3A). ΔMD for water was also different between stable and active groups (stable mean ΔMD = 0.05 μm^2^/ms, active mean ΔMD = − 0.03 μm^2^/ms, p = 0.02), driven by an average increase in water MD values at month 6 in stable cases (p = 0.03) (Fig. 3B). Brain volume was different between stable and active groups (stable MS mean Δ%ICV = − 0.01, active MS mean Δ%ICV = − 0.13, *p* = 0.04; Fig. 3C). On the other hand, water fractional anisotropy (Fig. 3D) did not demonstrate detectable changes over the 6-month study period in either stable or active MS. Δ**D**_cytosol_ and ΔMD were not correlated with disease duration (*ns*, Supplementary Fig. 2B).

## 4 Discussion

This longitudinal study, performed on a clinical 3 T MRI scanner with standard manufacturer-provided hardware, supports the hypothesis that diffusivity measurements of the intraneuronal metabolite *N*-acetylaspartate may reflect the health of axons in the corpus callosum in MS. The cross-sectional NAA diffusivity data presented here are in agreement with those from a prior DW-MRS study of MS that was performed on a research 7 T scanner and utilized different diffusion-weighting parameters and a different analysis scheme (Wood et al., 2012). In that cross-sectional study, the tensor-derived NAA parallel diffusivity in the corpus callosum was lower in MS compared to HVs, a finding confirmed in the present data. Additionally, in the current study we measured NAA diffusivity in the MS corpus callosum at baseline and month 6. We found that in cases with recent or ongoing disease activity, cytosolic NAA diffusivity in the corpus callosum decreased at month 6 compared to baseline, even though water mean diffusivity and fractional anisotropy in the same VOI did not change during this same period. These DW-MRS data were collected on 3 T clinical scanners in a scan time of 15–20 min per volume of interest, which was added to a scanning session that provided clinical T_1_-weighted images without and with contrast, FLAIR, and DTI.

### 4.1 Reproducibility and power

The reproducibility of Δ**D**_cytosol_ measured with this DW-MRS technique has been demonstrated previously (Wood et al., 2015). As such, we chose our sample size based on power calculations using this b-value scheme and number of averages; specifically, we expected that a sample size of 6–13 subjects per group would be sufficient to detect a between-group difference of 10–15%. Here, we found a difference of 14% in Δ**D**_cytosol_ between stable and active groups. The cytosolic NAA diffusivity values measured here are in line with previous reports in HVs (Ronen et al., 2014, Ronen et al., 2013, Wood et al., 2015) and experiments utilizing very short diffusion times (Marchadour et al., 2012, Najac et al., 2016). Analysis of the empirical apparent diffusion coefficient (ADC) of NAA without modeling yielded similar patterns of NAA diffusivity results, though without statistical significance. Additionally, the increased water diffusivity measures in MS compared to HVs (Bammer et al., 2000, Reich et al., 2010, Werring et al., 1999) and brain volume loss in MS (Fisher et al., 2008, Gao et al., 2014) are consistent with previous studies.

### 4.2 Possible relationship to underlying axonal pathology

Both the cross-sectional and longitudinal in vivo NAA diffusivity measurements reported here follow the pattern of changes expected for axonal degeneration. Models of molecular diffusion in cylinders (Landman et al., 2010) predict that water diffusivity, particularly parallel to the long axis of axons/cylinders (λ _||_), will decrease in configurations that occur in the setting of axonal degeneration (e.g. bulging, broken, or crimped cylinders). Histopathological studies of degenerating axons have demonstrated a variety of cytosolic changes, including alterations in neurofilament phosphorylation, mitochondrial breakdown with release of reactive oxygen species and decreased ATP production, and dissolution of intracellular enzymatic micro-compartments (Campbell and Mahad, 2011, Carroll, 2009, Petzold et al., 2011, Petzold et al., 2008). In principle, all of these changes could decrease the **D**_cytosol_ due to viscosity and molecular crowding.

A subset of DTI studies in MS and related animal models have demonstrated decreased water λ _||_. The commonality among this group is the timing of imaging in relation to clinical symptoms or inciting events. Animal studies, where the timing of pathology provocation is precisely known and serial imaging is the norm, allow histopathological verification. These studies consistently show early decreased water λ _||_, corresponding to histological evidence of axonopathy, followed by normalized or increased water λ _||_ and increased water λ _⊥_ when inflammation, demyelination, and gliosis become more prevalent (Budde et al., 2009, DeBoy et al., 2007, Loy et al., 2007, Sun et al., 2006, Xie et al., 2010). For instance, the dorsal root axotomy is an experimental procedure for studying Wallerian degeneration in which degenerating ascending fibers can be studied throughout their course in the spinal cord with comparison to the intact contralateral fibers. Study of this system at 11.7 T, with ex vivo high-resolution diffusion-weighted MRI analyzed by both conventional DTI and *q*-space DWI methods (Farrell et al., 2010, Zhang et al., 2009), demonstrated decreased water λ _||_ at 3 and 30 days post-axotomy. These changes were seen in conjunction with histological evidence of axon injury and degeneration, such as neurofilament phosphorylation state and the presence of myelin ovoids. This pattern of DTI changes is also borne out in the human epilepsy and optic neuritis studies that benefitted from a known time course due to surgical intervention and discrete clinical onset, respectively (Concha et al., 2006, Naismith et al., 2012). Since cerebral MS lesions are often silent, imaging studies are usually done without knowledge of, or respect to, their onset. Therefore, axonopathy is always found in a mixed setting of inflammation, edema, demyelination, and remyelination.

In the present study, we took advantage of the known increased rate of axonopathy seen in active inflammatory MS, hypothesizing that we would observe decreasing **D**_cytosol_ over a 6-month period. This hypothesis was borne out, but interestingly we also found that the annualized rate of change of water MD in the corpus callosum in the whole MS group was 0.13% (Fig. 3B). Relative to acute lesions, chronic lesions usually demonstrate a paucity of frank inflammation but may still be associated with ongoing demyelination and neurodegeneration, which can be especially pronounced in progressive MS (Frischer et al., 2009, Frischer et al., 2015, Lassmann, 2014). These processes might nonspecifically increase water MD in a centrally located and well-connected brain region such as the corpus callosum.

### 4.3 Limitations

Further assessment in a larger cohort over a longer duration could clarify whether our results are generalizable. At present, generalizability is limited by the relatively small sample size of both patients and HVs, as well as aspects of the study design. Due to the evolution of several initially stable MS cases into the active group, the groups became unbalanced, with only 6 MS cases remaining in the stable group. Additionally, we did not scan the spinal cord, which means that we might have missed new lesions even in the stable cases. The location and characteristics of lesions in the active group, and the treatment status in both groups, were heterogeneous. Scanner stability was not assessed over time. There were no month-6 scans for HVs (the HVs were scanned twice within 2 weeks), which means that HV data cannot be used as reference for the longitudinal progression of neurodegenerative processes but only as a marker of reproducibility of the method.

To partially address these limitations, data collected from anterior and posterior corpus callosum voxels were merged. This merging may have introduced regional and inter-individual biases. Mixed-effects regression was employed to mitigate this possible effect on the statistical models. Additionally, the anterior and posterior VOI data were examined separately in initial analyses. Overall, the separate VOIs demonstrated similar overall trends but were not statistically significant on their own. Although it will require further confirmation, the fact that VOI placement is not severely restricted with this technique may be a strength, though care should be taken to ensure that fiber direction within each VOI remains relatively homogeneous.

In general, MR spectroscopy measurements, and especially DW-MRS data, are limited by a low metabolite concentration compared to water. This means that NAA diffusivity measurements have a low signal-to-noise ratio compared to DTI and are therefore less sensitive than water MRI for picking up disease-related changes. The use of cardiac gating and associated variability in TR times could lead to partial and variable T1 saturation. Additionally, the requirement for a large VOI, imposed by low signal-to-noise, puts DW-MRS at risk for partial-volume effects and make it a very challenging technique for assessment of other clinically eloquent WM tracts (e.g. corticospinal tract). On the other hand, detailed analysis of the reliability and reproducibility of our DW-MRS technique demonstrated that this study was adequately powered to detect the differences in **D**_cytosol_ found here (Wood et al., 2015).

### 4.4 Future directions

Our data suggest that **D**_cytosol_ is more specific for axon-related changes, and hence more sensitive to axonopathy, than its water counterparts. For instance, the ΔNAA **D**_cytosol_ values of the stable and active MS cases greatly overlap, even though the groups were completely segregated by the presence or absence of new T_2_ lesions. DTI was no better than DW-MRS at determining disease activity, implying that DW-MRS measurements must be evaluated in the context of other neuroimaging and clinical data. For instance, DW-MRS cannot – and should not – take the place of high-resolution and tract-based techniques, such as DTI, which can provide whole brain and whole tract measures of WM structure. For instance, in a recent paper, Caligiuri et al. (2015) found that relatively specific measures of clinical progression (EDSS vs. Cognitive testing) were better correlated with changes in DTI measures localized to functionally related CC fibers than to measures of atrophy in those regions of the CC. They suggest that DTI measurements may be detecting an earlier “marker of fiber damage that has not yet resulted in macroscopic atrophy.”(Caligiuri et al., 2015). DWS contributes complementary information to this approach. Therefore, future studies should continue to investigate the relationship between these measures.

## 5 Conclusion

In summary, DW-MRS is a powerful technique that yields biologically interpretable data regarding the downstream effects of acute inflammatory demyelination and that can be applied in a clinical setting. Through its compartmental specificity, DW-MRS of NAA may provide answers to questions that are difficult to address with more conventional MRI techniques.

## Funding

This study was supported by the Intramural Research Program of the National Institute of Neurological Disorders and Stroke (grant number Z01 NS003119). ETW is the recipient of an NINDS Competitive Intramural Graduate Fellowship award.

## Appendix A Supplementary data

Supplementary figures Image 1

## References

[bb0005] Aboitiz F., Montiel J. (2003). One hundred million years of interhemispheric communication: the history of the corpus callosum. Braz. J. Med. Biol. Res..

[bb0010] Bammer R., Augustin M., Strasser-Fuchs S., Seifert T., Kapeller P., Stollberger R., Ebner F., Hartung H.P., Fazekas F. (2000). Magnetic resonance diffusion tensor imaging for characterizing diffuse and focal white matter abnormalities in multiple sclerosis. Magn. Reson. Med..

[bb0015] Banich M.T. (1998). The missing link: the role of interhemispheric interaction in attentional processing. Brain Cogn..

[bb0020] Bazin P.-L., Pham D.L. (2008). Homeomorphic brain image segmentation with topological and statistical atlases. Med. Image Anal..

[bb0025] Bergsland N., Horakova D., Dwyer M.G., Dolezal O., Seidl Z.K., Vaneckova M., Krasensky J., Havrdova E., Zivadinov R. (2012). Subcortical and cortical gray matter atrophy in a large sample of patients with clinically isolated syndrome and early relapsing-remitting multiple sclerosis. Am. J. Neuroradiol..

[bb0030] Bermel R.A., Bakshi R. (2006). The measurement and clinical relevance of brain atrophy in multiple sclerosis. Lancet Neurol..

[bb0035] Budde M.D., Xie M., Cross A.H., Song S.K. (2009). Axial diffusivity is the primary correlate of axonal injury in the experimental autoimmune encephalomyelitis spinal cord: a quantitative pixelwise analysis. J. Neurosci..

[bb0040] Caligiuri M.E., Barone S., Cherubini A., Augimeri A., Chiriaco C., Trotta M., Granata A., Filippelli E., Perrotta P., Valentino P., Quattrone A. (2015). The relationship between regional microstructural abnormalities of the corpus callosum and physical and cognitive disability in relapsing–remitting multiple sclerosis. Neuroimage Clin.

[bb0045] Campbell G.R., Mahad D.J. (2011). Mitochondrial changes associated with demyelination: consequences for axonal integrity. MITOCH 1–7.

[bb0050] Carass A., Cuzzocreo J., Wheeler M.B., Bazin P.-L., Resnick S.M., Prince J.L. (2011). Simple paradigm for extra-cerebral tissue removal: algorithm and analysis. NeuroImage.

[bb0055] Carroll S.L. (2009). Wallerian Degeneration.

[bb0060] Choi J.-K., Dedeoglu A., Jenkins B.G. (2007). Application of MRS to mouse models of neurodegenerative illness. NMR Biomed..

[bb0065] Ciccarelli O., Wheeler-Kingshott C., McLean M., Cercignani M., Wimpey K., Miller D., Thompson A. (2007). Spinal cord spectroscopy and diffusion-based tractography to assess acute disability in multiple sclerosis. Brain.

[bb0070] Concha L., Gross D.W., Wheatley B.M., Beaulieu C. (2006). Diffusion tensor imaging of time-dependent axonal and myelin degradation after corpus callosotomy in epilepsy patients. NeuroImage.

[bb0075] Davie C.A., Barker G.J., Thompson A.J., Tofts P.S., McDonald W.I., Miller D.H. (1997). 1H magnetic resonance spectroscopy of chronic cerebral white matter lesions and normal appearing white matter in multiple sclerosis. J. Neurol. Neurosurg. Psychiatry.

[bb2005] De Stefano N., Giorgio A., Battaglini M., Rovaris M., Sormani M.P., Barkhof F., Korteweg T., Enzinger C., Fazekas F., Calabrese M., Dinacci D., Tedeschi G., Gass A., Montalban X., Rovira A., Thompson A., Comi G., Miller D.H., Filippi M. (2010). Assessing brain atrophy rates in a large population of untreated multiple sclerosis subtypes.. Neurology.

[bb0080] DeBoy C.A., Zhang J., Dike S., Shats I., Jones M., Reich D.S., Mori S., Nguyen T., Rothstein B., Miller R.H., Griffin J.T., Kerr D.A., Calabresi P.A. (2007). High resolution diffusion tensor imaging of axonal damage in focal inflammatory and demyelinating lesions in rat spinal cord. Brain.

[bb0085] Evangelou N., Esiri M.M., Smith S., Palace J., Matthews P.M. (2000). Quantitative pathological evidence for axonal loss in normal appearing white matter in multiple sclerosis. Ann. Neurol..

[bb0090] Farrell J.A.D., Zhang J., Jones M.V., DeBoy C.A., Hoffman P.N., Landman B.A., Smith S.A., Reich D.S., Calabresi P.A., van Zijl P.C.M. (2010). q-Space and conventional diffusion imaging of axon and myelin damage in the rat spinal cord after axotomy. Magn. Reson. Med..

[bb0095] Filippi M., Bozzali M., Rovaris M., Gonen O., Kesavadas C., Ghezzi A., Martinelli V., Grossman R.I., Scotti G., Comi G. (2003). Evidence for widespread axonal damage at the earliest clinical stage of multiple sclerosis. Brain.

[bb0100] Fink F., Klein J., Lanz M., Mitrovics T., Lentschig M., Hahn H.K., Hildebrandt H. (2010). Comparison of diffusion tensor-based tractography and quantified brain atrophy for analyzing demyelination and axonal loss in MS. J. Neuroimaging.

[bb0105] Fisher E., Lee J.-C., Nakamura K., Rudick R.A. (2008). Gray matter atrophy in multiple sclerosis: a longitudinal study. Ann. Neurol..

[bb0110] Frischer J.M., Bramow S., Dal-Bianco A., Lucchinetti C.F., Rauschka H., Schmidbauer M., Laursen H., Sorensen P.S., Lassmann H. (2009). The relation between inflammation and neurodegeneration in multiple sclerosis brains. Brain.

[bb0115] Frischer J.M., Weigand S.D., Guo Y., Kale N., Parisi J.E., Pirko I., Mandrekar J., Bramow S., Metz I., Bruck W., Lassmann H., Lucchinetti C.F. (2015). Clinical and pathological insights into the dynamic nature of the white matter multiple sclerosis plaque. Ann. Neurol..

[bb0120] Gao K.C., Nair G., Cortese I.C.M., Koretsky A., Reich D.S. (2014). Sub-millimeter imaging of brain-free water for rapid volume assessment in atrophic brains. NeuroImage.

[bb0125] Ge Y., Gonen O., Inglese M., Babb J.S., Markowitz C.E., Grossman R.I. (2004). Neuronal cell injury precedes brain atrophy in multiple sclerosis. Neurology.

[bb0130] Gonen O., Catalaa I., Babb J.S., Ge Y., Mannon L.J., Kolson D.L., Grossman R.I. (2000). Total brain *N*-acetylaspartate: a new measure of disease load in MS. Neurology.

[bb0135] Hasan K.M., Walimuni I.S., Abid H., Datta S., Wolinsky J.S., Narayana P.A. (2012). Human brain atlas-based multimodal MRI analysis of volumetry, diffusimetry, relaxometry and lesion distribution in multiple sclerosis patients and healthy adult controls: implications for understanding the pathogenesis of multiple sclerosis and consolidation of quantitative MRI results in MS. J. Neurol. Sci..

[bb0140] Hasan K.M., Walimuni I.S., Abid H., Wolinsky J.S., Narayana P.A. (2012). Multi-modal quantitative MRI investigation of brain tissue neurodegeneration in multiple sclerosis. J. Magn. Reson. Imaging.

[bb0145] Inglese M., Li B.S.Y., Rusinek H., Babb J.S., Grossman R.I., Gonen O. (2003). Diffusely elevated cerebral choline and creatine in relapsing-remitting multiple sclerosis. Magn. Reson. Med..

[bb0150] Jenkinson M., Smith S. (2001). A global optimisation method for robust affine registration of brain images. Med. Image Anal..

[bb0155] Jones B.C., Nair G., Shea C.D., Crainiceanu C.M., Cortese I.C.M., Reich D.S. (2013). Quantification of multiple-sclerosis-related brain atrophy in two heterogeneous MRI datasets using mixed-effects modeling. Neuroimage Clin.

[bb0160] Kan H.E., Techawiboonwong A., van Osch M.J.P., Versluis M.J., Deelchand D.K., Henry P.-G., Marjańska M., van Buchem M.A., Webb A.G., Ronen I. (2012). Differences in apparent diffusion coefficients of brain metabolites between grey and white matter in the human brain measured at 7 T. Magn. Reson. Med..

[bb0165] Kappos L., Radue E.-W., O'Connor P., Polman C., Hohlfeld R., Calabresi P., Selmaj K., Agoropoulou C., Leyk M., Zhang-Auberson L., Burtin P., FREEDOMS Study Group (2010). A placebo-controlled trial of oral fingolimod in relapsing multiple sclerosis. N. Engl. J. Med..

[bb0170] Kirov I.I., Patil V., Babb J.S., Rusinek H., Herbert J., Gonen O. (2009). MR spectroscopy indicates diffuse multiple sclerosis activity during remission. J. Neurol. Neurosurg. Psychiatry.

[bb0175] Kirov I.I., Tal A., Babb J.S., Herbert J., Gonen O. (2012). Serial proton MR spectroscopy of gray and white matter in relapsing-remitting MS. Neurology.

[bb0180] Landman B.A., Farrell J.A.D., Smith S.A., Reich D.S., Calabresi P.A., van Zijl P.C.M. (2010). Complex geometric models of diffusion and relaxation in healthy and damaged white matter. NMR Biomed..

[bb0185] Lassmann H. (2014). Mechanisms of white matter damage in multiple sclerosis. Glia.

[bb0190] Leary S.M., Davie C.A., Parker G.J., Stevenson V.L., Wang L., Barker G.J., Miller D.H., Thompson A.J. (1999). 1H magnetic resonance spectroscopy of normal appearing white matter in primary progressive multiple sclerosis. J. Neurol..

[bb0195] Lenzi D., Conte A., Mainero C., Frasca V., Fubelli F., Totaro P., Caramia F., Inghilleri M., Pozzilli C., Pantano P. (2007). Effect of corpus callosum damage on ipsilateral motor activation in patients with multiple sclerosis: a functional and anatomical study. Hum. Brain Mapp..

[bb0200] Loy D.N., Kim J.H., Xie M., Schmidt R.E., Trinkaus K., Song S.K. (2007). Diffusion tensor imaging predicts hyperacute spinal cord injury severity. J. Neurotrauma.

[bb0205] Lucas B.C., Bogovic J.A., Carass A., Bazin P.-L., Prince J.L., Pham D.L., Landman B.A. (2010). The Java Image Science Toolkit (JIST) for rapid prototyping and publishing of neuroimaging software. Neuroinformatics.

[bb0210] Marchadour C., Brouillet E., Hantraye P., Lebon V., Valette J. (2012). Anomalous Diffusion of Brain Metabolites Evidenced by Diffusion-weighted Magnetic Resonance Spectroscopy In Vivo.

[bb0215] McAuliffe M.J., Lalonde F.M., McGarry D., Gandler W., Csaky K., Trus B.L. (2001). Medical Image Processing, Analysis and Visualization in clinical research, Proceedings 14th IEEE Symposium on Computer-Based Medical Systems CBMS 2001, 2001.

[bb0220] Naismith R.T., Xu J., Tutlam N.T., Lancia S., Trinkaus K., Song S.K., Cross A.H. (2012). Diffusion tensor imaging in acute optic neuropathies: predictor of clinical outcomes. Arch. Neurol..

[bb0225] Najac C., Branzoli F., Ronen I., Valette J. (2016). Brain intracellular metabolites are freely diffusing along cell fibers in grey and white matter, as measured by diffusion-weighted MR spectroscopy in the human brain at 7 T. Brain Struct. Funct..

[bb0230] Narayana P.A., Wolinsky J.S., Rao S.B., He R., Mehta M., PROMiSe Trial MRSI Group (2004). Multicentre proton magnetic resonance spectroscopy imaging of primary progressive multiple sclerosis. Mult. Scler..

[bb0235] Oh J., Pelletier D., Nelson S. (2004). Corpus callosum axonal injury in multiple sclerosis measured by proton magnetic resonance spectroscopic imaging. Arch. Neurol..

[bb0240] Ozturk A., Smith S.A., Gordon-Lipkin E.M., Harrison D.M., Shiee N., Pham D.L., Caffo B.S., Calabresi P.A., Reich D.S. (2010). MRI of the corpus callosum in multiple sclerosis: association with disability. Mult. Scler..

[bb0245] Pelletier D., Nelson S.J., Oh J., Antel J.P., Kita M., Zamvil S.S., Goodkin D.E. (2003). MRI lesion volume heterogeneity in primary progressive MS in relation with axonal damage and brain atrophy. J. Neurol. Neurosurg. Psychiatry.

[bb0250] Petzold A., Gveric D., Groves M., Schmierer K., Grant D., Chapman M., Keir G., Cuzner L., Thompson E.J. (2008). Phosphorylation and compactness of neurofilaments in multiple sclerosis: indicators of axonal pathology. Exp. Neurol..

[bb0255] Petzold A., Tozer D.J., Schmierer K. (2011). Axonal damage in the making: neurofilament phosphorylation, proton mobility and magnetisation transfer in multiple sclerosis normal appearing white matter. Exp. Neurol..

[bb0260] Polman C.H., Reingold S.C., Banwell B., Clanet M., Cohen J.A., Filippi M., Fujihara K., Havrdova E., Hutchinson M., Kappos L., Lublin F.D., Montalban X., O'Connor P., Sandberg-Wollheim M., Thompson A.J., Waubant E., Weinshenker B., Wolinsky J.S. (2011). Diagnostic criteria for multiple sclerosis: 2010 revisions to the McDonald criteria. Ann. Neurol..

[bb0265] Provencher S.W. (1993). Estimation of metabolite concentrations from localized in vivo proton NMR spectra. Magn. Reson. Med..

[bb0270] Reich D.S., Ozturk A., Calabresi P.A., Mori S. (2010). Automated vs. conventional tractography in multiple sclerosis: variability and correlation with disability. NeuroImage.

[bb0285] Ronen I., Ercan E., Webb A. (2013). Axonal and glial microstructural information obtained with diffusion-weighted magnetic resonance spectroscopy at 7 T. Front. Integr. Neurosci..

[bb0290] Ronen I., Budde M., Ercan E., Annese J., Techawiboonwong A., Webb A. (2014). Microstructural organization of axons in the human corpus callosum quantified by diffusion-weighted magnetic resonance spectroscopy of *N*-acetylaspartate and post-mortem histology. Brain Struct. Funct..

[bb0295] Roosendaal S.D., Geurts J.J.G., Vrenken H., Hulst H.E., Cover K.S., Castelijns J.A., Pouwels P.J.W., Barkhof F. (2009). Regional DTI differences in multiple sclerosis patients. NeuroImage.

[bb0300] Rovaris M., Gallo A., Falini A., Benedetti B., Rossi P., Comola M., Scotti G., Comi G., Filippi M. (2005). Axonal injury and overall tissue loss are not related in primary progressive multiple sclerosis. Arch. Neurol..

[bb0305] Rudick R.A., Lee J.-C., Nakamura K., Fisher E. (2009). Gray matter atrophy correlates with MS disability progression measured with MSFC but not EDSS. J. Neurol. Sci..

[bb0310] Sajja B.R., Narayana P.A., Wolinsky J.S., Ahn C.W., PROMiSe Trial MRSI Group (2008). Longitudinal magnetic resonance spectroscopic imaging of primary progressive multiple sclerosis patients treated with glatiramer acetate: multicenter study. Mult. Scler..

[bb0315] Samann P.G., Knop M., Golgor E., Messler S., Czisch M., Weber F. (2012). Brain volume and diffusion markers as predictors of disability and short-term disease evolution in multiple sclerosis. Am. J. Neuroradiol..

[bb0320] Schirmer L., Antel J.P., Brück W., Stadelmann C. (2011). Axonal loss and neurofilament phosphorylation changes accompany lesion development and clinical progression in multiple sclerosis. Brain Pathol..

[bb0325] Shiee N., Bazin P.-L., Ozturk A., Reich D.S., Calabresi P.A., Pham D.L. (2010). A topology-preserving approach to the segmentation of brain images with multiple sclerosis lesions. NeuroImage.

[bb0330] Shiee N., Bazin P.-L., Zackowski K.M., Farrell S.K., Harrison D.M., Newsome S.D., Ratchford J.N., Caffo B.S., Calabresi P.A., Pham D.L., Reich D.S. (2012). Revisiting brain atrophy and its relationship to disability in multiple sclerosis. PLoS One.

[bb0335] Sled J.G., Zijdenbos A.P., Evans A.C. (1998). A nonparametric method for automatic correction of intensity nonuniformity in MRI data. IEEE Trans. Med. Imaging.

[bb0340] Smith S.M., Zhang Y., Jenkinson M., Chen J., Matthews P.M., Federico A., De Stefano N. (2002). Accurate, robust, and automated longitudinal and cross-sectional brain change analysis. NeuroImage.

[bb0345] Stromillo M.L., Giorgio A., Rossi F., Battaglini M., Hakiki B., Malentacchi G., Santangelo M., Gasperini C., Bartolozzi M.L., Portaccio E., Amato M.P., De Stefano N. (2013). Brain metabolic changes suggestive of axonal damage in radiologically isolated syndrome. Neurology.

[bb0350] Suhy J., Rooney W., Goodkin D., Capizzano A., Soher B., Maudsley A., Waubant E., Andersson P., Weiner M. (2000). ^1^H MRSI comparison of white matter and lesions in primary progressive and relapsing-remitting MS. Mult. Scler..

[bb0355] Sun S.-W., Liang H.F., Trinkaus K., Cross A.H., Armstrong R.C., Song S.K. (2006). Noninvasive detection of cuprizone induced axonal damage and demyelination in the mouse corpus callosum. Magn. Reson. Med..

[bb0360] Tiberio M., Chard D.T., Altmann D.R., Davies G., Griffin C.M., McLean M.A., Rashid W., Sastre-Garriga J., Thompson A.J., Miller D.H. (2006). Metabolite changes in early relapsing-remitting multiple sclerosis. A two year follow-up study. J. Neurol..

[bb0365] Trapp B.D., Peterson J., Ransohoff R.M., Rudick R., Mörk S., Bø L. (1998). Axonal transection in the lesions of multiple sclerosis. N. Engl. J. Med..

[bb0370] Upadhyay J., Hallock K., Erb K., Kim D., Ronen I. (2007). Diffusion properties of NAA in human corpus callosum as studied with diffusion tensor spectroscopy. Magn. Reson. Med..

[bb0375] Wattjes M.P., Harzheim M., Lutterbey G.G., Klotz L., Schild H.H., Träber F. (2007). Axonal damage but no increased glial cell activity in the normal-appearing white matter of patients with clinically isolated syndromes suggestive of multiple sclerosis using high-field magnetic resonance spectroscopy. AJNR Am. J. Neuroradiol..

[bb0380] Werring D.J., Clark C.A., Barker G.J., Thompson A.J., Miller D.H. (1999). Diffusion tensor imaging of lesions and normal-appearing white matter in multiple sclerosis. Neurology.

[bb0385] Wood E.T., Ronen I., Techawiboonwong A., Jones C.K., Barker P.B., Calabresi P., Harrison D., Reich D.S. (2012). Investigating axonal damage in multiple sclerosis by diffusion tensor spectroscopy. J. Neurosci..

[bb0390] Wood E.T., Ercan A.E., Branzoli F., Webb A., Sati P., Reich D.S., Ronen I. (2015). Reproducibility and optimization of in vivo human diffusion-weighted MRS of the corpus callosum at 3 T and 7 T. NMR Biomed..

[bb0395] Xie M., Tobin J.E., Budde M.D., Chen C.-I., Trinkaus K., Cross A.H., McDaniel D.P., Song S.K., Armstrong R.C. (2010). Rostrocaudal analysis of corpus callosum demyelination and axon damage across disease stages refines diffusion tensor imaging correlations with pathological features. J. Neuropathol. Exp. Neurol..

[bb0400] Yaldizli O., Glassl S., Sturm D., Papadopoulou A., Gass A., Tettenborn B., Putzki N. (2011). Fatigue and progression of corpus callosum atrophy in multiple sclerosis. J. Neurol..

[bb0405] Zhang J., Jones M., DeBoy C.A., Reich D.S., Farrell J.A.D., Hoffman P.N., Griffin J.W., Sheikh K.A., Miller M.I., Mori S., Calabresi P.A. (2009). Diffusion tensor magnetic resonance imaging of Wallerian degeneration in rat spinal cord after dorsal root axotomy. J. Neurosci..

