# Supplementary material for: Longitudinal MR spectroscopy of neurodegeneration in multiple sclerosis with diffusion of the intra-axonal constituent N-acetylaspartate
Source: Neuroimage Clin. 2017 Jun 22;15:780–8. doi: 10.1016/j.nicl.2017.06.028 (PMC5496488; doi:10.1016/j.nicl.2017.06.028)

**Supplementary Figure 1. Lesion Load. A**. NAA **D**_cytosol_ is negatively correlated with lesion load (%WM) (*RC* = -0.02, *R*^2^ = 0.16, **p* < 0.05). **B**. Water fractional anisotropy is negatively correlated with lesion load (*RC* = -0.02, *R^2^ = 0.19, **p < 0.01*). **C**. Water mean diffusivity is positively correlated with lesion load (*RC* = 0.04, *R*^2^ = 0.18, **p* < 0.05). **D**. Water perpendicular diffusivity is positively correlated with lesion load (*RC* = 0.05, *R*^2^ = 0.19, ***p* < 0.01).

**Supplementary Figure 2.** **MS disease duration.** **A**. Disease duration for stable and active MS cases, not significant (ns), p = 0.14. **B**. Change in water mean diffusivity over 6 months versus disease duration (stable = open symbol, active = closed symbol), ns, p = 0.15.


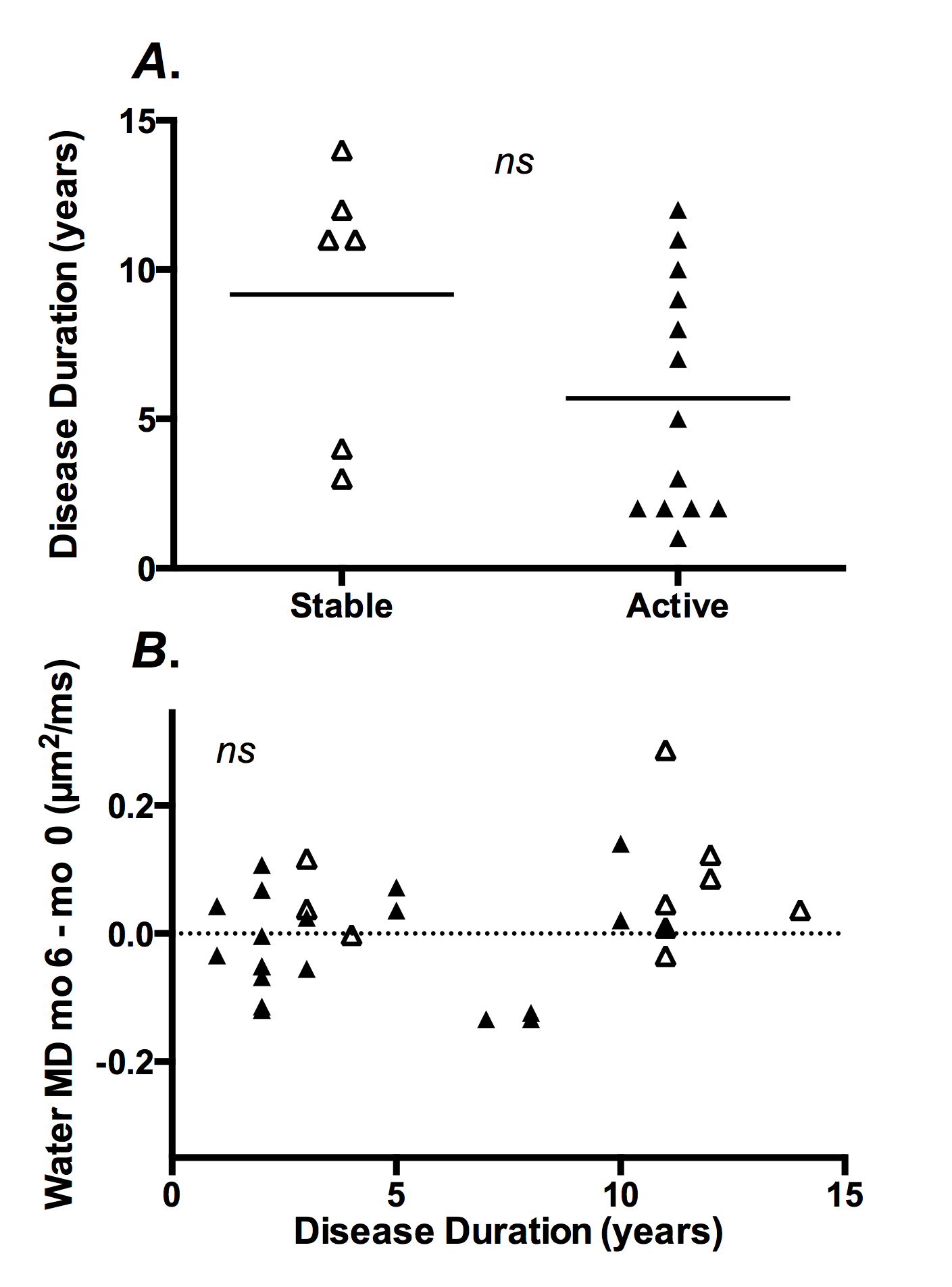

Supplement: Supplementary file 1 — Supplementary figures [file mmc1.docx]
